# Books Reviewed

**Published:** 1863-06

**Authors:** 


### BOOKS REVIEWED.

*Obstetrics, the Science and the Art.* By CHARLES D. MEIGS, M. D., *lately Professor of Midwifery and the Diseases of Women and Children in Jefferson Medical College at Philadelphia, and one of the Physicians to the lying-in department of the Pennsylvania Hospital; Member of the Society of Swedish Physicians; at Stockholm, corresponding member of the Hunterian Society of London; Member of the American Philasophical Society; of the Academy of Natural Sciences of Philadelphia; of the American Medical Association, &c., &c. Fourth edition, revised,* WITH ONE HUNDRED AND TWENTY-NINE ILLUSTRATIONS.—*Philadelphia:* BLANCHARD & LEA.

This book comes to us containing full and complete instruction for every condition of disease or accident pertaining to pregnancy and child-birth. The work is most admirably arranged, every chapter, section and paragraph being placed with great care and good taste. The book is published in the very best style of the art, and is a credit and ornament to a work of such intrinsic merit. It is hardly necessary or proper that we should speak of the essential composition and material of the book itself. The long time it has been known by the profession, and the high place it has attained as a standard work on obstetrics precludes the necessity of further comment. The world-wide reputation of the author also renders any expression of approbation of this book unnecessary. It is a full and complete treatise upon obstetrics, with numerous very fine illustrations, the whole comprising one of the best and most highly approved practical works. As for the changes and improvements of this edition we will allow the author to speak for himself.

"In this edition I have endeavored to amend the work by changes in its form; by careful corrections of many expressions, and by a few omissions and some additions to the text. The *student* will find that I have recast the article on Placenta Praevia, which I was led to do out of my desire to notice certain new modes of treatment which I regarded as not only ill founded as to the philosophy of our department, but dangerous to the people.

In changing the form of my work by dividing it into paragraphs or sections, numbering from 1 to 959, I thought to present to the reader a common-place book of the whole volume. Such a table of contents ought to prove both convenient and useful to the student while attending public lectures, whatsoever may be the title of his text-book on midwifery, since it refers seriatim to a great variety of topics on midwifery, the mere reference to which might well serve to recall a whole—even a long train of thoughts upon the subject under his review."

The division of the book into paragraphs is certainly a great improvement, and the Index and Table of Contents are very full indeed, and add greatly to the value of the book, especially for the actively engaged physician who uses such a work mainly as a book of reference.

Upon the point of revision of the chapter upon Placenta Previa, we desire to say a word, explaining a little more clearly the nature of the notice taken of the new modes of treatment for Placenta Previa by Drs. Radford or Thompson, at least mainly advocated by them. It consists in what is supposed to be a detaching of the placenta from its place on the womb, with a view to put an end to the unavoidable hemorrhage. "They are yet in bonds of the Hunterian dogma of the placental structure, and believe that the placenta consists of two distinct products, one called the fœtal portion, and the other the uterine or maternal portion of the placenta. Now, inasmuch as the progress of discovery in these matters has made it clearly known, that the uterus furnishes no portion of the placenta, and that that body is a fleshy, vascular excrescence, formed upon the exterior surface of the chorion, any hypothesis of practice or methodus medendi that is based upon the exploded notion of Mr. Hunter should be considered dissipated with that very explosion."

We are sorry we cannot copy at greater length the remarks of our author upon this subject. We must close our brief notice of a valuable book by quoting in brief his own plan for treatment of placenta prævia:

"Therefore one of the prime indications is to get her out of her trouble as soon as possible, with due regard to prudence. In any such case the physician ought to deliver by the feet if they should present, and if the head or any other part should present, his duty is to turn and deliver by the feet. Hence when a student asks what is to be done in ,his case of placenta prævia, I invariably reply, turn and deliver by the feet, for if the child does not present by the pelvic pole, the indication is certainly to turn and deliver by the feet."

"However, turning cannot be attempted until the os is sufficiently (not dilated) *dilatable* to allow the hand to pass inwards to take the feet. I trust the student will never attempt to force an undilatable os, and I am equally confident that no wise and prudent man will set himself down patiently to wait for dilatation. It is not dilatation that he is to expect, but dilatability, two ideas that are widely sundered as the poles. Let him therefore touch from time to time, so that he may know when the precious moment of dilatability is come; this is a thing that no man can do who stupidly resorting to the tampon shuts out all the light of diagnosis. I was on the very point of losing a most interesting woman in such a labor by this very piece of stupidity, but I have been better educated since that early day, and at the present day we are all far better off as to our resources than our fathers were, for we are in possession of the colpeurynter and the knowledge of its uses, which enables us to make the womb dilatable, and to so dilate it in about four hours as to admit of the passing a hand in search of the feet. Let every person, therefore, who is liable to be called upon in a case of implantation be sure his colpeurynter is not only at hand, but in good order."

---

*The Institutes of Medicine.* BY MARTYN PAINE, A. M., M. D., L. L. D. *Professor of the Institutes of Medicine and Materia Medica in the University of the City of New York; Corresponding Member of the Royal Verein Fur Helkunde in Preussen; Corresponding Member of the Royal Medico-Chirurgical Academy of Turin; Corresponding Member of the Gessellschaft Fur Natur and Heilkunde Zu Dresden; Member of the Medical Society at Leipsic; of the Medical Society of Sweden; of the Montreal Natural History Society; and of many other learned Societies. Seventh edition. New York:* HARPER & BROTHERS, *Publishers, Franklin Square,* 1862. *London:* LAMPSON, LOW, SON & Co., *American and European Booksellers,* 47 *Ludgate Hill.*

This book, upon the Institutes of Medicine, is doubtless familiar to many of our readers. It has long been regarded as a standard work upon the philosophy of medicine and merited the high estimation in which it has always been held. It shows that great labor has been bestowed in preparing it for the profession. The views of other authors have been carefully obtained and fully given upon many points, so that the work cannot be regarded as expressing alone the opinions of one man, but as comprehending all that is valuable, together with the opinions of the distinguished author. The philosophy of medicine—that is of physiology, pathology and therapeutics, is quite too little studied, and practitioners are liable thus to become experimenters, rather than careful students of medicine. The philosophy of therapeutics has not yet been made the careful study of the general practitioner to an extent which its importance demands; physicians have looked earnestly for new weapons, and new uses for old ones, but have too little considered the objections and dangers attending the employment of all active medication in the treatment of diseases—the philosophy of therapeutics is yet to be learned. Nearly all the agents used in the treatment of disease, together with the conditions and indications for their employment should be most carefully considered, or we shall follow a blind routine and become empirics. Physiology, pathology and therapeutics are rapidly undergoing revision; a wide field is here open for cultivation. Those interested in these subjects will find a vast amount of valuable information collected and embodied in the work under consideration. It is earnestly recommended as reliable, eminently practical, and every way worthy the confidence it receives.

---

